# Barriers and facilitators to self-care in chronic heart failure: a meta-synthesis of qualitative studies

**DOI:** 10.1186/2193-1801-2-320

**Published:** 2013-07-16

**Authors:** Soraya Siabani, Stephen R Leeder, Patricia M Davidson

**Affiliations:** Victor Coppleson Building - D02, Menzies Center for Health Policy, Medical School, The University of Sydney, Sydney, Australia; School of Health, Kermansha University of Medical Sciences, Kermanshah, Iran; Centre for Cardiovascular & Chronic Care, Faculty of Health, University of Technology, Sydney, Australia

**Keywords:** Congestive heart failure, CHF, Self-management, Treatment adherence, Compliance, Self-care determinants, Qualitative review

## Abstract

Chronic heart failure (CHF) is a costly condition that places large demands on self-care. Failure to adhere with self-care recommendations is common and associated with frequent hospitalization. Understanding the factors that enable or inhibit self-care is essential in developing effective health care interventions. This qualitative review was conducted to address the research question, "What are the barriers and facilitators to self-care among patients with CHF?” Electronic databases including Medline, EMBASE, CINAHL, Web of Science, Scopus and Google scholar were searched. Articles were included if they were peer reviewed (1995 to 2012), in English language and investigated at least one contextual or individual factor impacting on self-care in CHF patients > 18years. The criteria defined by Kuper et al. including clarity and appropriateness of sampling, data collection and data analysis were used to appraise the quality of articles. Twenty-three articles met the inclusion criteria. Factors impacting on self-care were included factors related to symptoms of CHF and the self-care process; factors related to personal characteristics; and factors related to environment and self-care system. Important factors such as socioeconomic situation and education level have not been explored extensively and there were minimal data on the influence of age, gender, self-confidence and duration of disease. Although there is an emerging literature, further research is required to address the barriers and facilitators to self-care in patients with CHF in order to provide an appropriate guide for intervention strategies to improve self-care in CHF.

## Introduction

Chronic heart failure (CHF) is a chronic progressive condition where the heart fails to meet the body’s metabolic demands. CHF is an increasingly common and burdensome illness especially among older people and is a major cause of mortality, morbidity and poor quality of life worldwide (Go et al. 2013; AIHW 2011). The prevalence of CHF has been reported as 0.4% to 2% in the general population and between 2.3% to over 16% among those aged >75years (Go et al. 2013; AIHW 2011; Heidenreich et al. 2011; Anguita Sanchez et al. 2008; Masoudi et al. 2002). A large proportion of health care resources increasingly goes towards treating cardiovascular diseases (CVD), especially CHF. Many of these costs are attributed to hospitalization (Go et al. 2013; Berry et al. 2001; Chen et al. 2010). Hospitalization for CHF exacerbations could often be prevented by care plans considering self-care as a core for their health programs (Klersy et al. 2011; Ditewig et al. 2010; Hertzog et al. 2010). Furthermore, effective self-care has been critical in promoting optimal outcomes in CHF (Chen et al. 2010; Grady 2008) and reducing mortality rates (Ditewig et al. 2010).

Self-care in CHF is described as a naturalistic decision-making process enabling engagement with healthy behaviors such as daily monitoring and adherence to the plan of care (self-care maintenance), and adequate management of symptoms and evaluation of applied treatment actions (self-care management) (Riegel et al. 2009; Moser et al. 2012). Despite the advantages of self-care, patients with CHF, especially older people, face numerous difficulties in achieving optimal quality of self-care (Powell et al. 2008; Muzzarelli et al. 2010; Sayers et al. 2008).

Appreciating the factors that enable or inhibit self-care is critical in developing effective recommendations for self-care. However, there is no comprehensive study which clarifies these factors. For this purpose, reviewing qualitative studies is important in understanding patients’ experiences (Dixon-Woods et al. 2005; Thorne et al. 2004; Barnett-Page & Thomas 2009), health seeking behaviours (Murray 1998) and providing a clear insight into barriers and facilitators through uncovering beliefs and motivations of individuals (Dixon-Woods et al. 2005; Kent & Fineout-Overholt 2008). The aim of this meta-synthesis review (Sandelowski & Barroso 2003) was to review studies that identify barriers and facilitators to self-care in patients with CHF.

## Method and subjects

### Search strategy

A meta-synthesis of qualitative studies was undertaken to address the research question; what are the facilitators or barriers to self-care in patients with CHF? Electronic data bases of Medline, Embase, CINAHL, Web of Science, Scopus and Google scholar were systematically searched for articles. A combination of MeSH terms and text words, under supervision of a health librarian, was used to explore each database. To increase the sensitivity of the search strategy and avoid missing valuable studies, a wide range of possible terms for self-care were used in combination with MeSH terms for chronic heart failure (e.g. heart failure and self-care). The most key words, terms and subject headings were; self-management, self-maintenance, self-monitoring, self-regulation, help-seeking, adherence , compliance, daily weight monitoring, sodium limitation, fluid restriction, sign, symptom, behavior, barriers, facilitators, help-seeking, physical activity and exercise.

### Inclusion criteria and Quality assessment

**Figure 1:**
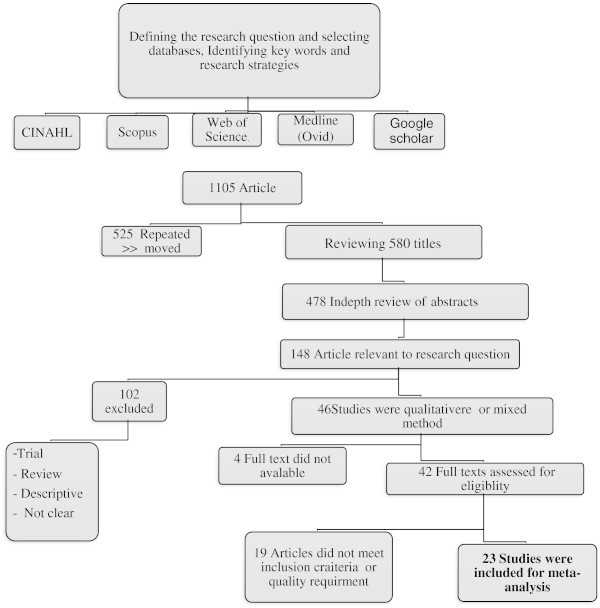
**The flow chart for selecting qualitative articles on facilitators and barriers of self-care in patients with CHF.**

Qualitative studies were included if they were peer-reviewed and published between1995 and June 2012. This timeframe was chosen because it corresponded to an upsurge in the discourse on self-management. Studies were in the English language and investigated at least one contextual or individual factor impacting on self-care in patients with CHF > 18years. The wide variety of qualitative methods made it difficult to compare and critique such research (Sandelowski & Barroso 2003; Sandelowski & Emden 1997). The absence of commonly applicable criteria to use in critically appraising these papers, as might be used in a systematic review of quantitative studies with regard to the sample size, process of randomization, and assessment of statistical significance limited the critical process (Solomon 2009). Of available guidelines for this purpose (Kuper & Levinson 2008; CG 2010; Collingridge & Gantt 2008; Kitto et al. 2008), criteria from (Kuper & Levinson 2008) were used (Table 1). Appraising the paper from (Falk et al. 2007), by way of example, is presented in Table 1. Based on these criteria, studies were ranked as very good, good, acceptable or unclear (Table 2, organized chronologically) Articles that had clear research aims and met at least four of the six appraisal criteria from (Kuper & Levinson 2008) were included. Two criteria; *possibility of applying the results of study in other settings* and *addressing potential ethical issues* were not taken into consideration as exclusion criteria because the generalizability of results emerging from qualitative studies is controversial (Myers 2000), and most article addressed ethical issues even if they did not explain details in reports, given the conventional requirement of most journals for evidence of ethical clearance of studies submitted for publication. Finally, Twenty-three articles were found to meet the inclusion criteria (Figure 1).

**Table 1:** **Criteria from Kuper & Levinson (**2008**) applied in appraising studies; a study cunducted by Falk et al. (**2007**) was appraised as an example**

Was what the researchers did clear?	Yes, researchers explicated aim, methodology (e.g. sampling, inclusion criteria, data gathering) results and analysis with a clear scenario.
Was the sample used appropriate to its research question?	Yes, it was a phenomenology study. Sampling has clearly been articulated. 17 patients living with CHF who had personal experiences about HF to address the research question. Sample size was broad enough to capture many aspects of the CHF. However, they did not acknowledge socioeconomic situation and other associated factors
Were the data collected appropriately?	Yes, setting had been justified for data collection. Data were collected through recorded interviewing that is appropriate for exploring experiences of stakeholders in a phenomenology. Question as “what do you mean?” was used that is fit for phenomenological studies. Data collection was done by nurses familiar with CHF and continued to achieve data saturation.
Were the data analyzed appropriately?	Yes, The study had a clear description of data analysis process. Subcategories, categories and themes were derived from data by separate interpreters and then compared and combined. Some quotas from original data were used for supporting findings.
Can the results of this study be transferred to other settings?	Researchers did not discuss transferring results to other populations; however, they gave recommendations for caregivers and patients in general.
Did the study adequately address potential ethical issues?	Yes, researchers had a comprehensive presentation of ethical issues including achieving approval from ethics committee and chief physician plus informing the participants about study followed by consent from them

**Table 2:** **Quality of studies included in a meta-synthesis of researches relating to self-care in patients with CHF (n = 23)**

Author /year / country / reference number	Study design /tools/population	Aim	Was what the researchers did clear?	Was the aim /research question clear?	Is the sample used appropriate to its research question?	Were the data collected appropriately?	Were the data analyzed appropriately?	Can the results of this study be transferred to other settings?	Does the study adequately address potential ethical issues?
Martensson et al. (1997) Sweden	Phenomenology/ Interview (n = 12 patients)	To investigate how patients conceive their life situation	G^**^	G	A^***^	VG^*^	G	U^****^	G
Rogers et al. (2000) United Kingdom	In-depth interview (n = 27 patients)	Investigate patients’ need for information	G	VG	G	G	A	U	U
Buetow et al. (2001) New Zealand	Narrative/ Semi-structured questionnaire/ Interview (n = 62 patients)	To illustrate how patients cope with their illness	G	VG	G	VG	G	A	U
Riegel & Carlson (2002) USA	Interview/ Structured questionnaire (n = 26 patients)	To explain better adaptation in some people	VG	VG	G	VG	G	A	G
Horowitz et al. (2004) USA	Grounded theory/ Semi-structured interview (n = 19 patients)	To elucidate patients’ belief and knowledge & understand factors underling self-care routines	VG	G	A	VG	G	U	G
Scotto (2005) USA	Phenomenology/ Interview (n = 14 patients)	To explore the experience of patients living with HF and their adherence to prescribed regimens	VG	VG	VG	G	G	A	G
Eldh et al. (2006) Sweden	Narrative/ Interview + observation (n = 4 patients + 2 Nurses)	To explore patients’ participation/non- participation in a CHF care program	G	G	A	G	VG	U	VG
Riegel et al. (2006) USA	Mixed method/ Interview (n = 15 patients)	To evaluate a motivational interviewing intervention and identify the mechanisms by which it influenced HF self-care	G	VG	A	A	G	N^*****^	G
Rucker-Whitaker et al. (2006) USA	Focus group ( n = 25 patients)	To understand from the patient perspective what factors promote/limit retention in a self-management improvement program	A	G	A	A	A	N	U
Schnell et al. (2006) Canada	Semi-structured interview (n = 11 patients)	To explore self-care experience living with CHF	G	A	G	G	G	G	G
Falk et al. (2007) Sweden	Phenomenology/Interview (n = 17)	To describe how persons living with CHF perceived the maintenance of their daily life	VG	G	G	VG	VG	U	U
Davidson et al. (2007) Australia	Interview (triangulation study) (n = 17 patients +13 family + 16 key-informants) + literature	To describe health patterns, information needs, and adjustment process for overseas-born people with heart failure living in Australia	G	A	G	VG	G	A	VG
Riegel et al. (2007) USA	Mixed method/ Interview (n = 29 patients)	To describe how expertise in CHF self-care develops	G	VG	VG	G	G	G	U
Kaholokula et al. (2008) USA	Focus group (n = 11 patients +25 nurses)	To describe health beliefs, attitudes, practices and social and family relations important in CHF treatment	VG	VG	G	G	G	N	G
Rodriguez et al. (2008) USA	Grounded theory/ Semi-structured telephone interview (n = 25 patients)	To explore patients’ knowledge about CHF diagnosis and their understanding of cardiac care providers’ recommendations	A	G	A	A	G	N	VG
Sheahan & Fields (2008) USA	Semi-structured questionnaire/ Focus group (n = 33 patients)	To explore factors associated with sodium-restricted diet	A	G	G	G	G	G	VG
Dickson et al. (2008) USA	Mixed method/ Semi-structured interview (n = 41 patients)	To identify the influences of attitudes and self-efficacy on HF self-care management	VG	VG	VG	G	G	G	G
Clark et al. (2009) USA	Semi-structured interview (n = 42 patients + 30 informal caregiver)	To explore factors (perceived by patients and health givers) influencing willingness and capacity to manage CHF	G	VG	G	G	A	G	VG
Dickson & Riegel (2009) USA	Qualitative descriptive meta- analysis of their 3 studies (n = 85 patients)	To assess self-care skill in CHF patients and explore their skill needs	G	G	G	G	A	U	U
Granger et al. (2009) USA	Open-ended questionnaire/ In-depth interview (n = 6 patients and 6 physicians)	To explore patients’ and their physicians’ perspectives about adherence and how the exchange of information between them is experienced by each group	G	G	A	A	G	U	VG
Riegel et al. (2010a) USA	Mixed method/ Interview/ Open-ended question (n = 27)	To describe CHF self-care in men and women and to identify gender-specific barriers and facilitators influencing CHF self-care	VG	VG	A	VG	G	N	VG
Ming et al. (2011) Malaysia	Semi-structured interview (n = 20 patients)	To explore patients’ experiences of self-management and identify factors influencing patients’ adherence to medications	G	G	G	G	G	G	VG
Dickson et al. (2012) published online) USA	Mixed method/ Interview/Open-ended question (n = 30)	To describe the cultural beliefs about self-care, identify social factors influencing self-care and how these factors influence self-care practices	VG	G	G	G	G	G	G

* VG = Very good **G = Good ***A = Acceptable ****U = Unclear *****N = No.

This study was a part of research that has been approved by the ethics committee of the University of Sydney.

## Findings

Participants in the reviewed studies included 477 patients (289 male and 188 female) between 38 to 98 year old livings with CHF. Most studies included men and women with the exception of Martensson et al. 1997; who selected only 12 males and Sheahan & Fields 2008who had an exclusively female sample (n = 33). Time since diagnosis of CHF in eight studies was a minimum of six months (Falk et al. 2007; Schnell et al. 2006; Clark et al. 2009; Ming et al. 2011; Dickson et al. 2012; Riegel et al. 2010b), while other investigators included cases with a diagnosis duration of one month or more. Six studies did not report New York Heart Association (NYHA) functional classification of their participants (Buetow et al. 2001; Scotto 2005; Rucker-Whitaker et al. 2006; Davidson et al. 2007; Kaholokula et al. 2008; Sheahan & Fields 2008). Most other investigators included patients with functional classes II, III and IV. However, about 75% of participants were in advanced stages of CHF, classes III or IV (Martensson et al. 1997; Buetow et al. 2001; Horowitz et al. 2004; Riegel et al. 2006; Rucker-Whitaker et al. 2006; Riegel et al. 2007). Seven studies (Riegel & Carlson 2002; Scotto 2005; Riegel et al. 2006; Rucker-Whitaker et al. 2006; Schnell et al. 2006; Riegel et al. 2007; Sheahan & Fields 2008) reported one or more comorbidities (e.g. diabetes) in their subjects. The participants of three studies included doctors or nurses (as careers, not as patients) along with patients and their families (Table 2).

In Table 3, where articles were organized chronologically, the important findings of facilitators and barriers to self-care in patients with CHF that emerged from the 23 studies were summarized. Results showed that atypical and puzzling symptoms of CHF, complexity of the self-care process, insufficient knowledge, comorbidity burden, cognitive decline and memory loss, depression and anxiety, poor communication skills, adverse coping strategies (avoidance and denial) hinder self-care in patients with CHF. On the other hand, supportive environments, disavowal coping strategy, trust in health care providers, spiritual beliefs and optimism were identified as positive contributing factors to self-care in these patients. The role of personal values, cultural issues (Martensson et al. 1997; Dickson et al. 2012) and acceptance (Riegel & Carlson 2002) were controversial in different studies, where some reported they made a positive contribution and others found a negative contribution to CHF self-care. In general, barriers and facilitators fell into three categories: a. factors related to the *symptoms of CHF and self-care processes*; b. factors related to *personal characteristics*; and c. *environmental and health care givers’* factors.

**Table 3:** **Barriers and facilitators to self-care in chronic heart failure**

Author / Year/ Country / Ref	Barriers /and the behaviours they affected	Facilitators/ and the behaviors they affected
Martensson et al. (1997) Sweden	- Physical limitation, feeling lack of energy / physical activity	- Awareness of threat / physical activity
	- Hopelessness / decision making and motivation for management symptom	- Environmental support/ self-confidence
	- Short term memory loss and confusion / taking medicinel	
(Rogers et al. 2000) United Kingdom	- Misconception about CHF / medical and regimen adherence	
	- Acceptance / decision making	
	- Lack of facility / access to medical care	
	- Avoidance, acceptance and denial / to obtain new information for caring themselves, and participate in decision making	
Buetow et al. (2001) New Zealand	- Multiple medicine, side effects of medicine / adherence to treatment	- Coping strategies of disavowal / taking medication and following prescriptions
Riegel & Carlson (2002) USA	- Lack of knowledge / adherence to regimen and exercise	- Supportive strategies; emotionally and tangibly / motivation, hope, adaptation with CHF
	- Atypical symptom and complexity of symptom / failing in following recommended diet	
	- Negative emotion and no environment support/ motivation	
	- Comorbidity / complexity of self-care and difficulty in symptom recognition	
	- Inadequate information (about CHF, its symptoms and their management)/ symptom recognition and definition of source of exacerbation symptom + symptom monitoring + receiving medical care	
Horowitz et al. (2004) USA		
Scotto (2005) USA	- Conflict between values of patients and nurses / not accepting new information and recommendation	- Acceptance and support from health care professional / adaptation to new life leads to adherent to appropriate self-care behaviors ( physical activity and adherence to prescribed instructions)
Eldh et al. (2006) Sweden	- Insufficient knowledge of educators and nurses / patients’ knowledge and skill for self-care	- Respect for patients / Increasing their knowledge and Participate in decision making
Riegel et al. (2006) USA	- Lack of knowledge / specially regarding diet and salt restriction	- Sympathy, reflective listening, acknowledging cultural values / engage patients to enhance their knowledge, skill and motivation to fallow self-care rules
		- Information / building skills of self-care in patients -Stimulating supporting resources / collaboration and participate in care programs
Rucker-Whitaker et al. (2006) USA	- Denial and anxiety / taking medicine	- Social activity and mutual support / motivation
		- Education especially with patients’ own language / adherence to regimen
	Dissatisfaction with received care / failed perceived benefit of self-care action such salt limitation	
Schnell et al. (2006) Canada	Hopelessness / motivation for physical activity and dietary regimen	- Social support, satisfaction with health system delivery/Positive outlook, perform self-care behavior
		- Simplicity of self-care/ daily weighing and symptom monitoring,
		- Understanding reason for self-care / perceived health care roles, perceived benefit associated with physical activity
Falk et al. (2007) Sweden	Cultural issues, health seeking behaviours / adherence to regimen	- Trust family and formal care givers / following instructions
		- Social activity/ physical activity
		- perceiving the risk of withdrawing medicine / adherence to medicine and regimen
		- Facility ( Care availability) / care - seeking
Davidson et al. (2007) Australia	- Cognition problems due to CHF symptom / weighting, regiment, taking water pill	
Riegel et al. (2007) USA	- Depression / motivation for self-care	
	- Poor family functioning / self-care maintenance and management	
	- Denial of illness/ adherence to regimen and treatment	
Kaholokula et al. (2008) USA	- Hopelessness/ decision making	
	- Lack of family knowledge/ misconception about treatment preference	
	- Financial -burden / adherence to regimen	
	- Lack of trust physicians / medical using herbal medicine	
	- Lack of information about CHF symptom / symptom recognition and help-seeking	
Rodriguez et al. (2008) USA	- Comorbidity / symptom recognition (confusion about cause of symptoms )	
	- Lack of knowledge / sodium restriction and decision making	
Sheahan & Fields (2008) USA	- Loneliness/ motivation to care	- Living with family / motivation for adherence to regime
	- Cultures / dietary behaviours	
	- Lack of experience / medical adherence and symptom recognition	
Dickson et al. (2008) USA	- Side effect of medicine and interfere in work and normal life / medical adherence	- Long time experiences of HF / self-management and symptom monitoring
	Traditional education & insufficient skill in educators / developingself- maintenance (Diet, diuretic titration and exercise , low salt diet)	
Dickson & Riegel (2009) USA	- Complexity of self-care rules and no agreement between doctors and patients about this difficulty, hopelessness / fitting prescribed regimen into daily life all aspects of self-care were affected)	
Granger et al. (2009) USA	- Side effects of medicine interfering with social activities / medical adherence	
Clark et al. (2009) USA	- HF symptoms/ symptom recognition	
	- Lack of knowledge / self-management e.g. help-seeking and	
	- Lack of confidence / self-management	
	- Personal values linked to culture/ help-seeking	
	- Female, depression/ self-care confidence, decision making and interpreting symptoms	
Riegel et al. (2010a) USA	- Poor family support/ symptom –management	- Male/ self-care confidence and symptom recognition
		- Family support , hopefulness/ symptom management
	- Complexity medicine / adherence to medicine	
Ming et al. (2011) Malaysia	- Limited communication of doctors / adherence to medication	Family support/ self-care confidence and adherence to treatment
	- Difficulty in remembering/ adherence to medication and regimen	
	- Cultural issues / adherence to diet (having favorite food)	
Dickson et al. (2012/ published online) USA	- Knowledge /symptom monitoring and management (e.g. attributing CHF to stress)	- Social support / adherence to regimen and self- confidence
		- Financial support and access to facilities / adherence to medication,
		- Spirituality / motivate to care for themselves
		- Some cultural belief leading to strong familial support / engaging in self-care maintenance and self-care management, e.g. preparing unsalted food by family.

### Facilitators and barriers related to CHF syndrome and the process of self-care

Buetow et al. reported that recognizing CHF symptoms, especially atypical symptoms such as dizziness, fatigue, sleepiness, cognitive decline and loss of consciousness, was difficult for patients with CHF (Buetow et al. 2001). Patients found it difficult to interpret (Ming et al. 2011) or respond (Granger et al. 2009) to complex symptoms, especially in combination. Horowitz et al. reported that patients found difficulty either in recognizing or responding to symptoms in an exacerbation (Horowitz et al. 2004). Furthermore, suffering from such symptoms reduced the ability of patients to engage in efficacious self-care (Clark et al. 2009; Granger et al. 2009). Even patients who had sufficient knowledge about HF and self-care frequently were unable to manage exacerbations of HF symptoms (Falk et al. 2007; Horowitz et al. 2004; Scotto 2005; Riegel et al. 2006). Also, functional limitation and dependency linked to CHF have been reported as serious barriers to self-care in patients with advanced HF (classes III and IV) by three studies (Martensson et al. 1997; Riegel & Carlson 2002; Granger et al. 2009).

Complexity of self-care processes and difficulty in adhering to dietary change was another barrier to self-care in patients living with CHF (Riegel & Carlson 2002; Sheahan & Fields 2008; Granger et al. 2009). Following a diagnosis of CHF, the prescribed actions can seem complex and require situational skills (Dickson & Riegel 2009). Granger et al. and Dickson et al. found that physicians and patients have differing perceptions of instructions and that as a result patients were at times unable to understand and apply what their doctor instructed them to do. (Granger et al. 2009; Dickson et al. 2012)

### Facilitators and barriers related to personal characteristics

The lack of knowledge of CHF patients, especially regarding diet and salt restriction, and misconceptions about CHF and its symptoms leading to failure of understanding of the relationship between disease and symptoms, were prominent themes as barriers to self-care in the reviewed studies (Riegel & Carlson 2002; Horowitz et al. 2004; Eldh et al. 2006; Rodriguez et al. 2008; Sheahan & Fields 2008; Ming et al. 2011). Patients identified health awareness and understanding the consequences of ignoring the treatment plan and indicators of a worsening condition as facilitator stimulating care for themselves (Falk et al. 2007; Martensson et al. 1997; Schnell et al. 2006). Regarding duration of CHF, only one study reported that patients with long- standing CHF fared better than those recently diagnosed (Dickson et al. 2008). Using a disavowal coping strategy helped patients to affirm their physical health (Martensson et al. 1997; Buetow et al. 2001). However, using avoidance and denial reduced the capability of patients to care for themselves (Buetow et al. 2001; Riegel & Carlson 2002; Eldh et al. 2006; Rucker-Whitaker et al. 2006; Kaholokula et al. 2008). Acceptance had two contradictory consequences; as a barrier (Falk et al. 2007; Buetow et al. 2001; Riegel & Carlson 2002) and as a facilitator (Buetow et al. 2001; Riegel & Carlson 2002; Scotto 2005). Also, depression (Riegel & Carlson 2002; Riegel et al. 2007), anxiety (Rucker-Whitaker et al. 2006) and hopelessness were found to be negative factors for self-care maintenance, self-care management and self-care confidence, while positive belief in the future could serve as a catalyst for self-care (Falk et al. 2007; Martensson et al. 1997; Kaholokula et al. 2008).

According to the results, cultural beliefs and personal values might lead to a misguided conception of CHF (Dickson et al. 2012), difficulty with adherence to a healthy diet (Sheahan & Fields 2008; Dickson et al. 2012), preventing help-seeking (Clark et al. 2009), non-adherence to recommendations and health messages (Eldh et al. 2006; Kaholokula et al. 2008). On the other hand, cultural beliefs and personal values may support some aspects of self-care such as medication adherence (Dickson et al. 2012).

The influence of gender on self-care was explored by only one study (Riegel et al. 2010a). Although males and females exhibited equal medical adherence (self-care maintenance), females had lower self-care confidence and engaged less in self-care management and showed less accurate symptom interpretation than males (Riegel et al. 2010a). However, males had stronger social support than women and more positive perspectives on their lives and their ability to perform a self-care role than women (Riegel et al. 2010a). Cognitive decline and short term memory loss were important barriers to adherence to treatment and self-car plans especially in older people with CHF (Riegel & Carlson 2002; Granger et al. 2009; Ming et al. 2011; Dickson et al. 2012). Age as a direct influencing factor was not reported by the reviewed studies, though it was referred when speaking about disabilities and comorbidities (Riegel & Carlson 2002).

### Facilitators and barriers related to environment and health care givers

Supportive environments, either mutual from other patients or from family, neighbours, nurses and physicians facilitated self-care in terms of self-care confidence, adaptation with disease and reducing anxiety, adherence to treatment and food regimen, symptom management, positive outlook, and motivation to obtain information and care for own selves (Falk et al. 2007; Martensson et al. 1997; Riegel & Carlson 2002; Scotto 2005; Riegel et al. 2006; Schnell et al. 2006; Ming et al. 2011; Dickson et al. 2012) and poor self-care was associated with poor family support (Riegel et al. 2007; Riegel et al. 2010a). Also, respecting patients (Schnell et al. 2006) and acknowledging their values encouraged them to follow health regimens (Eldh et al. 2006; Riegel et al. 2006). In contrast, poor communication skill of care givers (Ming et al. 2011) and lack of trust health care professionals (Clark et al. 2009) prevented patients from seeking information required for self-care. Applying traditional teaching methods (Dickson & Riegel 2009) and not spending sufficient time with patients to train them about self-care (Kaholokula et al. 2008) or a failure to teach them practically (Granger et al. 2009) were other reported problems.

## Discussion

This review identified factors influencing self-care in patients with CHF. However, only CHF symptoms and comorbidity, knowledge, environmental support, and psychological factors are discussed here due to their frequency.

### Symptoms of chronic heart failure and comorbidities; complexity of symptom recognition

In recognizing and managing their symptoms, patients with CHF faced many difficulties and problems. First, confusing symptoms of CHF and cognitive impairment, especially in elderly patients, may reduce individuals' mental ability to recognize their symptoms and develop effective symptom management as a core for self-care (Ming et al. 2011). Second, illness severity may limit the capacity of a patient to manage symptoms. As an example, exhaustion and shortness of breath limited the individual’s ability not only for engaging in daily physical activity recommended in self-care instructions (e.g. 30minutes exercise daily) (Riegel et al. 2009) but stopped them managing when an exacerbation of CHF occurs (Granger et al. 2009; Ming et al. 2011). Third, symptoms might be attributed to other health problems and patients became confused in seeking to distinguish the reason and origin of their symptoms. In such situations, patients failed to take the correct action. As an example patients with CHF suffering, from arthritis might became confused about whether their swollen ankles are due to heart failure or arthritis (Riegel et al. 2009).

Furthermore, functional limitation may be compounded by other chronic problems such as forgetfulness, memory loss, diabetes, arthritis and chronic obstructive pulmonary disease in elderly patients (Martensson et al. 1997; Riegel & Carlson 2002; Riegel & Carlson 2002; Clark et al. 2009). Complex collections of problems produced frustration and feelings of hopelessness for patients, their families and even caregivers (Rogers et al. 2000; Riegel et al. 2007; Granger et al. 2009). It also is not easy for these patients to adhere to medical prescriptions and diet regimes (Dickson et al. 2012).

### Insufficient knowledge

Misconception due to insufficient knowledge was associated with self-care challenges in all aspects including medical and dietary adherence, (Rogers et al. 2000; Rucker-Whitaker et al. 2006), weighing (Riegel & Carlson 2002), symptom recognition (Horowitz et al. 2004), treatment performance (Kaholokula et al. 2008) and help-seeking (Rodriguez et al. 2008). Although these results were not unexpected findings (Field et al. 2006), it elucidated and supported many other studies that considered knowledge a critical foundation for self-care in patients with CHF (Toman et al. 2001; Artinian et al. 2002; Bushnell 1992; Wright et al. 2003; Miche et al. 2003). Insufficient information not only directly prohibited patients from understanding and applying instructions, but also introduced other barriers to self-care in patients with CHF (Kaholokula et al. 2008; Clark et al. 2009; Dickson et al. 2012). For instance, consumption of salty foods recognized by some investigators as a behavior associated with cultural beliefs and/or social norms might simply be a manifestation of ignorance due to insufficient knowledge about the pathophysiology of CHF or the role of self-maintenance. Having sufficient knowledge and accurate information was necessary but not sufficient for behaviour change. Many patients were not able to incorporate their knowledge into their daily lives (Granger et al. 2009) because they were not equipped with practical skills (Eldh et al. 2006; Dickson & Riegel 2009).

Important factors associated with the failure of self-care programs included inadequate educational approach due to unskilled health educators (Eldh et al. 2006; Albert et al. 2002) or a gap in provider knowledge of self-care instructions (Lainscak et al. 2011), applying too general instruction for specific situations (Dickson et al. 2008) or inappropriate educational program-planning (Boren et al. 2009), lack of assessment of self-care educational programs (Lainscak et al. 2011). The majority of educational programs were short duration interventions without sufficient on-going support from providers to continue the programs and build self-care skills (Boren et al. 2009; De Lusignan et al. 2001; Sethares & Elliott 2004). Only half of patients were given comprehensive information about self-care monitoring and self-care maintenance (Boren et al. 2009). Considering the economic effectiveness of self-care (Jeon et al. 2009) researchers and health policymakers might well recommend applying more resources to evaluating specific programs for specific conditions of patients.

### Environmental factors and cultural beliefs; motivators and hinders

There is evidence that patients with CHF suffered from social isolation (Jeon et al. 2010) that is associated with higher mortality in CHF (Horne & Weinman 1999). Although Sayers et al. pointed out that social support is not strongly associated with better self-care (Sayers et al. 2008), a review study stated that social support was prognostic in patients with HF (Ka & Lip 2002). The results of the current study showed that a supportive environment is critical for creating positive feelings and improving virtually all self-care components in patients with CHF (Falk et al. 2007; Martensson et al. 1997; Riegel & Carlson 2002; Scotto 2005; Riegel et al. 2006; Schnell et al. 2006; Ming et al. 2011; Dickson et al. 2012). Patients who have an opportunity to share their problems and those who participate in social activities manifest better self-care (Falk et al. 2007; Rucker-Whitaker et al. 2006). By way an example, eating alone reduced one’s motivation to cook and share meals resulting in an increased consumption of ‘microwave dinners’ often with high sodium content (Sheahan & Fields 2008).

Cultural beliefs (another theme that emerged from this review) might give patients a misguided conception of CHF, leading them to think, for example, that CHF is due to stress or simply associated with old age (Dickson et al. 2012). As a result, patients attempted to overcome a stressful situation by not following medical instruction. In addition, cultural preferences (e.g. favourite foods) often caused difficulty with adherence to a healthy diet (e.g. high salt consumption) (Sheahan & Fields 2008). However, cultural beliefs and personal values may support some aspects of self-care such as medication adherence. Dickson and her colleagues reported that spirituality influences self-care positively (Dickson et al. 2012).

In order to manage contextual problems such as cultural issues, health providers and educators needed to have good communication skills including reflective listening, empathy and acknowledging patients’ personal values (Jeon et al. 2010; Gilbert & Hayes 2009). Effective communication skills and trust have a reciprocal relationship; by improving one the other will be strengthened (Halpert & Godena 2011; Thomas 2011). However, according our findings, poor doctor-patient communication was an important barrier to self-care in patients with CHF (Kaholokula et al. 2008; Ming et al. 2011). Horne and Weinman found that medication beliefs are a stronger predictor of adherence than sociodemographic factors and clinical situation (Kramer 2010). A lack of faith in health care professionals along with personal values and cultural beliefs may stop patients from seeking help when symptoms worsen because the symptoms were culturally perceived to be uncontrollable and have to be accepted stoically (Clark et al. 2009).

### Psychological factors

Evidence shows that depression in patients with CHF is much more prevalent than general population (MacMahon & Lip 2002). On the other hand, depression resulting in lack of energy leads to unwanted effects on self-care (Turner & Kelly 2000). In addition, depression may increase the risk of death in this group of patients (Horne & Weinman 1999). Whereas experiencing positive emotions allows people to engage in behaviours that protect their positive state (Frantz 2004). The current study indicated that hopelessness and depression was observed as a significant problem for patients with CHF, especially lonely women (Riegel et al. 2010a). This influenced self-care confidence, symptom recognition (self-care management), though, adherence to medicine (self-care maintenance) was not affected by level of depression (Riegel et al. 2010a). Providing circumstances in which patients may continue with their leisure activities and assisting patients to have a better quality of life can result in improved moods and self-care ability.

Patients with CHF facing stressful situations and changes in life circumstances, employ a range of defence mechanisms and coping strategies some enabling and others less so (Kramer 2010; Telford et al. 2006). Telford and colleagues found that common reactions of patients suffering from chronic diseases include *denial* then *acceptance* (Telford et al. 2006). The current review showed that *avoidance* and *denial* reduce the capability of patients to care for themselves (Riegel & Carlson 2002; Eldh et al. 2006; Rucker-Whitaker et al. 2006). *Disavowal* (palliating the emotional strain at the same time as affirming their health) assisted patients to cope with their mental stresses without ignoring the reality of their disease (Buetow et al. 2001). *Acceptance* is a coping strategy that had both positive (Falk et al. 2007; Buetow et al. 2001; Riegel & Carlson 2002) and negative (Buetow et al. 2001; Riegel & Carlson 2002; Scotto 2005) effects on different personality and living in various cultural contextual.

## Limitations

Although there was an emerging literature, only rarely did studies report using the classic qualitative methodologies such as ethnography, grounded theory, phenomenology etc. Consequently, categorization of studies (that usually facilitates both evaluating articles and also amalgamating and analyzing data comprehensively) was difficult. Also, many authors had reported little raw data. In addition, factors such as socioeconomic situation and education level were not explored extensively and there were minimal data on the influence of age, gender, and the role of specific comorbidities. Virtually nothing has been published on the value or otherwise of lay versus professional educators. Furthermore, facilitators of self-care were not investigated as much as barriers.

## Conclusion

This review indicates that self-care is a complex and multi-faceted phenomenon that needs a comprehensive consideration of patients including their emotional situation, psychological characters, physical abilities, family support, living facilities, comorbidities (especially cognitive function) and their ability for learning. Insufficient knowledge about CHF, symptom recognition and ways of self-care along with hopelessness and psychological problems limited their abilities for an effective self-care. A supportive environment, motivation and adequate care programs using effective educational methods that build self-care skills, should be recommended to health care providers and families. Nevertheless, further research is required to address the barriers and facilitators of self- care in patients with CHF.
